# Extended Effect of Chronic Social Defeat Stress in Childhood on Behaviors in Adulthood

**DOI:** 10.1371/journal.pone.0091762

**Published:** 2014-03-25

**Authors:** Irina L. Kovalenko, Anna G. Galyamina, Dmitry A. Smagin, Tatyana V. Michurina, Natalia N. Kudryavtseva, Grigori Enikolopov

**Affiliations:** 1 Institute of Cytology and Genetics SD RAS, Novosibirsk, Russia; 2 NBIC, Moscow Institute of Physics and Technology, Moscow, Russia; 3 Cold Spring Harbor Laboratory, Cold Spring Harbor, New York, United States of America; Roma Tre University, Italy

## Abstract

Individuals exposed to social stress in childhood are more predisposed to developing psychoemotional disorders in adulthood. Here we use an animal model to determine the influence of hostile social environment in adolescence on behavior during adult life. One-month-old adolescent male mice were placed for 2 weeks in a common cage with an adult aggressive male. Animals were separated by a transparent perforated partition, but the adolescent male was exposed daily to short attacks from the adult male. After exposure to social stress, some of the adolescent mice were placed for 3 weeks in comfortable conditions. Following this rest period, stressed young males and adult males were studied in a range of behavioral tests to evaluate the levels of anxiety, depressiveness, and communicativeness with an unfamiliar partner. In addition, adult mice exposed to social stress in adolescence were engaged in agonistic interactions. We found that 2 weeks of social stress result in a decrease of communicativeness in the home cage and diminished social interactions on the novel territory. Stressed adolescents demonstrated a high level of anxiety in the elevated plus-maze test and helplessness in the Porsolt test. Furthermore, the number of dividing (BrdU-positive) cells in the subgranular zone of the dentate gyrus was significantly lower in stressed adolescents. After 3 weeks of rest, most behavioral characteristics in different tests, as well as the number of BrdU-positive cells in the hippocampus, did not differ from those of the respective control mice. However, the level of anxiety remained high in adult males exposed to chronic social stress in childhood. Furthermore, these males were more aggressive in the agonistic interactions. Thus, hostile social environment in adolescence disturbs psychoemotional state and social behaviors of animals in adult life.

## Introduction

Upon exposure to social stress, adolescents are at a greater risk than individuals of other age groups to develop psychoemotional disorders, such as heightened anxiety or depression [1], [2], [3]. Therefore, possible effects of various psyhopathogenic factors, especially those of social nature originating in childhood, are the focus of a growing number of studies. Various animal models have been employed to demonstrate the effects of stress on adolescents. For instance, it has been shown that long-term social isolation can induce learning and memory disturbances, as well as increased levels of anxiety in young animals [4] and that in adolescent rats, inescapable tail shock stress reduces social exploration and activates the serotonergic dorsal raphe nucleus [5]. Stress-induced behavioral and physiological changes in adolescents can be long-lasting and persist into adulthood [6], [7]. For instance, in male rats, social instability stress alters cell proliferation in the hippocampal dentate gyrus in adolescence and produces deficits in spatial location memory in adulthood [8]; it also results in persistent alterations of hypothalamus-pituitary-adrenal axis function and increased anxiety [9]. Chronic restraint stress during adolescence affects basal corticosterone levels and decreases neurogenesis in the dentate gyrus of adult female rats [10], suggesting that stress during adolescence has long-term consequences for hypothalamic-pituitary-adrenal axis function and hippocampal plasticity in adulthood. Adult female rats that were isolated in adolescence exhibited behavioral changes in the forced swim test and showed increased preference for sucrose compared with adult females that were group-housed in adolescence [11]. Chronic mixed-modality stressor (consisting of isolation, restraint and social defeat stress) during adolescence has been shown to result in different and sustained changes leading to depressive-like behavior in rats: stressed animals display decreased sucrose consumption, hyperactivity in the elevated plus-maze, and decreased activity in the forced swim test during both adolescence and adulthood [12]. Social defeat in adolescence appears to increase attack latencies [13]. Chronic social stress induced by rotations in group compositions during adolescence induced cognitive dysfunction, such as substantial impairment of spatial memory in aged mice [14]. The majority of studies indicate that early-life stress can lead to a heightened stress response in maternally deprived rodents tested as adults [7]. Overall, chronic deprivation of early maternal care and also chronic deprivation of early physical interactions with conspecifics are profound risk factors for the development of inappropriate aggressive behaviors [15]. Taken together, there is convincing evidence that certain types of stress during adolescence can have long-lasting consequences and affect adult behavior. However, a number of studies on adolescent stress also emphasize high resilience of adolescents and their capacity to avoid developing long-lasting psychopathological changes in behavior after being exposed to stress (reviewed in [6]). Also, it has been shown that effects of early stress and its consequences may depend on species, sex and strain of animals [10], [11], [13], [16], [17].

Our study aims to determine the extended influence of hostile social environment in adolescence on behaviors in adulthood. As a chronic social stress to adolescent mice, we exposed them to residing in a common cage with an adult aggressive male, being separated by a transparent perforated partition. Additionally, the adolescents were exposed to chronic social defeat stress by lifting the partition for a short time to allow attacks by the adult aggressor mice. Our results show that hostile social environment in adolescence disturbs psychoemotional state and social behavior of animals in adult life.

## Materials and Methods

### Animals

Male mice of the C57BL/6J strain from a stock maintained in the Animal Facility of the Institute of Cytology and Genetics, SD RAS, (Novosibirsk, Russia) were used. The animals were housed under standard conditions (12∶12 hr light/dark regime, switch-on at 8.00 a.m.; food (pellets) and water available *ad libitum*). Experiments were performed on 4-week-old (adolescent) and 10–12-week-old (adult) mice. All procedures were in compliance with the European Communities Council Directive of November 24, 1986 (86/609/EEC). The study was approved by Scientific Council N 9 of the Institute of Cytology and Genetics SD RAS of March, 24, 2010, N 613.

### Experiment 1. Effects of Chronic Social Stress on the Behaviors of Adolescent Mice in Adolescence and Adult Life

Adult male mice (potential aggressors) were placed for 5 days into one of the two equal compartments of experimental cages separated by a transparent perforated partition. On the sixth day, single 4-week-old male adolescents were placed into the vacant compartments of common cages. Daily between 14∶00 and 17∶00, standard covers were replaced with transparent ones and 5 min later (period of activation) the partitions were removed. Given access, as a rule, all adult males demonstrated aggression toward adolescents. They attacked and chased the young males, which, in turn, demonstrated flight and defensive behavior. Agonistic interactions between the adult and adolescent males continued for 5 min or for less than 3 min if the attack by an adult male was intense, after which animals were separated. Every day, each young male was transferred into an unfamiliar cage next to another aggressive adult partner, living on its own territory. Such exposure of adolescent mice to social stress continued for 2 weeks. In a separate experiment, after 2 weeks of social stress the defeated adolescents were placed in comfortable conditions (in common cages with a friendly partner of the same age and social experience) for 3 weeks. Another group of adolescents was individually transferred daily into a compartment of a partitioned cage next to an unfamiliar adult male but was not allowed to communicate physically with adult males. Male mice of similar age living in littermate groups were used as controls. Before being examined in a range of behavioral tests, at the end of a period of rest, all animals were placed in individual cages to facilitate behavioral testing and remove group housing effects. Behaviors of male mice were studied consecutively in a “one test per day” regimen in the partition, elevated plus-maze, social interactions (“cylinder” test), open-field and Porsolt tests to estimate the level of communicativeness with unfamiliar partner in home and novel cages, anxiety, and depressiveness. Thus, four groups of adolescents were studied:

1. Control – age-matched males reared in littermate groups (environment without stress);
2. Social-defeat-stressed adolescents (SDS adolescents) – adolescents exposed for 2 weeks to daily aggression in agonistic interactions with unfamiliar adult males (hostile environment);
3. Psychologically stressed adolescents (PS adolescents) – adolescents that were placed next to an adult male but were deprived of physical contact (exposure regarded as a psychological stress) – communication deprivation stress (CDS);
4. Adult mice subjected to social defeat stress in adolescence following 3 weeks of rest in comfortable conditions.

**Figure 1:**
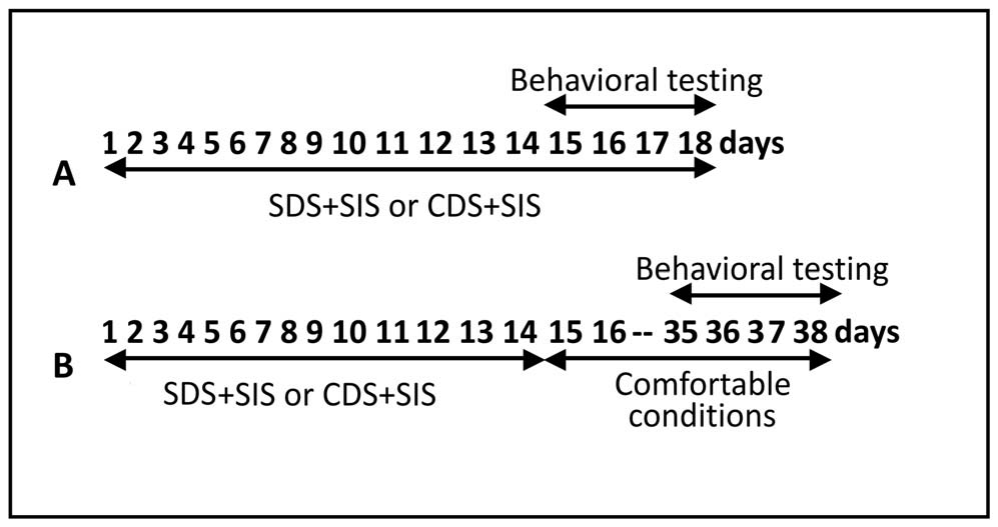
Protocols for studying the effects of combined stress (SDS+SIS or CDS+SIS) on the behavior of adolescents (A) and adult mice stressed in adolescence (B). SDS - social defeat stress; CDS – communication deprivation stress; SIS – social instability stress. As a control age-matched groups of males living in littermate groups were used. Before being examined in a range of behavioral tests, control animals and adult mice at the period of 31–38 days (B) were placed in individual cages to facilitate behavioral testing and remove group housing effects. Experimental groups and age-matched control groups were tested simultaneously in the behavioral tests (one test per day). Details of protocol are described in section “Materials and methods”.

It should be noted that in our experimental design, adolescents were daily subjected to a social instability stress (SIS), since for 2 weeks each young male was placed in an unfamiliar cage next to a different aggressive adult partner living on its own territory in a partitioned compartment. Thus, combinations of two stressors were considered as psychogenic factors: SDS+SIS for SDS adolescents and CDS+SIS for PS adolescents. Experimental protocols is presented in Fig. 1.

### Experiment 2. Agonistic Behavior of Adult Male Mice Subjected to SIS and SDS in Adolescence

Following a 3-week period of rest (living with male mice of the same age and social experience), adult male mice exposed to social stress in adolescence were subjected to an agonistic interaction test. Each male mouse was placed into one of the experimental cage compartments divided by a transparent perforated partition as described above. A group-housed male of the same age and weight was placed into the neighboring compartment to serve as a partner. Two days later the partition was removed and the agonistic interactions were videotaped. The males were marked according to the outcome of the first interaction as winners or losers. Adult male mice without negative social experience in adolescence studied in similar conditions were used as controls.

### Immunocytochemistry, Quantitation of Dividing Cells, Image Capture

Separate groups of animals were used for morphological studies. Adolescents exposed to social stress were studied on the day following the day of the last agonistic interaction with an adult male. After 3 weeks of rest, males and age-matched controls were also examined. Transcardial perfusion and brain sectioning were performed in accordance with standard protocols for tissue fixation and processing [18], [19]. BrdU was injected 2 hours before perfusion. Animals were deeply anesthetized with 3% Avertin (2, 2, 2-tribromoethanol, Sigma*-*Aldrich, St. Louis, MO) and then were subjected to transcardial perfusion with 30 mL of phosphate-buffered saline (PBS) followed by 30 mL of 4% paraformaldehyde (PF) in PBS, pH 7.4. The brains were removed and postfixed overnight with the same fixative at 4°C, then transferred to PBS with 0.1% sodium azide and kept at 4°C until sectioning. Before sectioning, brains were cut sagittally into two hemispheres. One brain hemisphere was randomly selected per animal and serial 50 μm thick sections were collected using Vibratome 1500 (Vibratome, St. Louis, MO). Brain sections from all experimental groups were processed simultaneously throughout all stages of the immunohistochemical procedure.

Immunostaining was carried out following standard protocols. Briefly, brain sections were denaturated in 2N HCl at 37°C for 1 hour. After neutralization in 0.1 M borate, sections were incubated with blocking and permeabilization solution (PBS containing 1% Triton-100X and 3% goat serum) for 2 hours at room temperature, and then incubated overnight at 4°C with the rat anti-BrdU primary antibody (1∶300, Accurate Chemical Inc., Westbury, NY) diluted in PBS containing 0.2% Triton-100X and 3% goat serum. After washing with PBS, the sections were incubated with fluorochrome-conjugated AlexaFluor 488 goat anti-rat secondary antibodies (1∶400, Molecular Probes, Eugene, OR) diluted in PBS containing 0.2% Triton-100X and 3% goat serum for 2 hours at room temperature. After washing with PBS, the sections were mounted on gelatin-coated slides with DakoCytomation Fluorescent Mounting Medium (DakoCytomation, Carpinteria, CA). For each mouse in each group, data were obtained from 7–9 sections containing dentate gyrus of the hippocampus. The dentate gyrus of the hippocampus was anatomically identified in accordance with the stereotaxic mouse brain atlas [20]. Quantitative analysis of BrdU-labeled cells was performed by epifluorescence microscopy.

### Behavioral Tests

#### Partition test

The partition test was used to estimate the behavioral response of mice to a conspecific [21]. Mice were placed into the experimental cage, with a transparent perforated partition dividing the cage into equal parts. The number of approaches to the partition and the total time spent near it (moving near the partition, smelling and touching it with one or two paws, clutching and hanging, putting noses into the holes or gnawing the holes) were scored during 5 min as indices of reacting to the partner. The time during which the males showed sideways position or were “turning away” near the partition was not included in the total time scored.

The experimental procedure was as follows: the adolescents and adult males were placed into separate compartments of a cage with a partition. On the testing day, behavioral responses of the adolescents toward the familiar partner were recorded for 5 min. Then a familiar partner was carefully replaced by an unfamiliar one (group-housed male) and behavior was further recorded for 5 min.

#### Elevated plus-maze test

The elevated plus-maze test [22] was conducted using a plus-maze consisting of two open arms (25×5 cm) and two closed arms (25×5×15 cm). The two arms of each type were opposite to each other and extended from a central platform (5×5 cm). The floor and side-walls of the maze were of gray opaque Plexiglas® material. The maze was elevated to a height of 50 cm above the floor. Five min before exposure to the plus-maze, the standard cover of the mouse-containing cage was replaced by a transparent cover in the same room. The mouse was placed at the center of the plus-maze with its nose to the closed-arm center. The following parameters of behavior were recorded during 5 min: 1) total entries; 2) open-arm entries (four paws in open arm), closed-arm entries (four paws in closed arm), and central platform entries; 3) time spent in open arms, closed arms, and central platform; 4) the number of passages from one closed arm to another; 5) the number of head dips (looking down toward the floor below the plus-maze); 6) the number of peepings when the mouse is in closed arms (mouse extends its head from the closed arm and returns quickly back). Indices 1 and 4 are related to locomotor activity; Indices 2 and 3 are considered as measures of the level of anxiety; Indices 5 and 6 are considered as parameters of risk assessment behavior [23]. The numbers of entries to the closed arms, open arms, and to the central platform were calculated as percentages of the total entries, and periods spent in the closed arms, open arms, and in the central platform were calculated as percentages of total testing time. The plus-maze was placed in a dimly lit room and thoroughly cleaned between sessions.

#### Social interaction test (“cylinder” test)

Animals were placed into the open-field (36×23 cm) Plexiglas arena with an upside-down perforated cylinder placed in one of the cage corners. Each mouse was then placed individually in the opposite corner for 5 min for adaptation to the new situation. After 5 min, an unfamiliar group-housed male was carefully placed under the cylinder for 5 min. Behavior of the animals was recorded when the cylinder was empty and when the partner was placed under the cylinder for 5 min each, and the data were documented. The following behavioral variables were registered: 1) the number of approaches to the cylinder and the total time(s) spent near it (moving near the cylinder, smelling and touching it with nose, one or more paws, getting on a cylinder and a contact with it by four paws) were scored as indices of reacting to the empty cylinder or an unfamiliar partner under the cylinder; 2) the number of instances of rearing; 3) the duration of self-grooming (licking of the fur on the flanks or abdomen, washing over the head from ear to snout). The time the males showed sideways position or “turning away” near the cylinder was not included in the total time. Between the sessions, the cages and cylinders were thoroughly washed with water and dried with napkins.

#### Open-field test

The open-field test was carried out in a 9×9 square blue painted 100×100 cm Plexiglas open field. It was illuminated by a 150 W electric lamp, 150 cm above the floor. Mice were placed individually in the center of the box and the following behavioral parameters were recorded for 5 min: 1) Latency of first movement from center (sec), 2) number of crossed squares, 3) number of fecal boluses (defecation) and 4) total time of self-grooming (sec). Between the sessions, the cages and cylinders were thoroughly washed with water and dried with napkins.

#### Porsolt test

[24]. Each male was placed in a glass beaker (16.5 cm height, 11 cm inner diameter) containing 10 cm of water at t = 25±1°C for a 5-min period. The total time of full immobility without any movements, total time of active avoidance (active behavior), as well as the time of drift (the time during which the mouse slowly moved around the beaker, moving one or two paws and supporting its body on the surface of the water) were recorded. The sum of drift time and full immobility time was recorded as passive behavior. Latency of first demonstration of full immobility during 5 sec (without any movement) was also recorded. Between the sessions, the water was changed in the glass beaker.

#### Agonistic interaction test

After 5 min of activation, partitions were removed and the behavior of animals in the agonistic interactions test was video-recorded for 10 min during its first encounter, and the data were documented. The following behavioral domains were analyzed for males that were stressed in adolescence, demonstrated aggressive behavior, and became the winners in agonistic interaction with the grouped male of the same weight and age: 1) Attacks: latency of the first attack, attacking, biting and chasing 2) Aggressive grooming: the winner mounting the loser’s back, holding it down and spending much time licking and nibbling at the scruff of the loser’s neck. During such aggressive grooming the loser appears fully immobilized, or sometimes stretches out its neck and then again freezes under the winner; 3) Digging: digging up and scattering the sawdust on the loser’s territory (kick-digs: pulling the sawdust forward with the forepaws; push-digs: pushing the sawdust backward with the hind paws); 4) Hostile behavior: the total time spent attacking, aggressively grooming and digging; 5) Self-grooming: body care activities (fur licking, head washing, nose washing); 6) Threats and rotations. The total time or number of events, as well as the fraction of animals demonstrating aggressive grooming, were measured. The Observer XT and the EthoVision software (Noldus Information Technology, the Netherlands) were used for analysis of animals’ behavior.

### Statistical Analysis

Normal distribution and homogeneity of variances were tested by the Shapiro-Wilk’s and Levene’s tests, respectively. ANOVA for repeated measures was used for the partition test with factor “groups” (control, SDS adolescents, PS adolescents), and factor “partner” (familiar partner, unfamiliar partner), and for the social interaction test with factor “partners” (empty cylinder, cylinder with tester). For other behavioral parameters in different tests, one-way ANOVA by ranks with factor “groups” was used. A *post hoc* pairwise comparison of the groups was made using the Bonferroni test or the LSD test (for the Porsolt test). For comparison of behaviors of animals stressed in adolescence after the 3 weeks of rest and the respective controls, the t-test was used. The U-test for nonparametric data was used for comparison of behavioral parameters in the test of agonistic interactions in this comparison. Percentage of animals demonstrating aggressive grooming was compared using the chi-square test. The data are reported as mean ± SEM (n = 10–13 per group). The statistical significance was set at P≤0.05, tendency at 0.05<P<0.1.

## Results

### Influence of Hostile Environment on the Behaviors of Adolescents Subjected to 2-week Chronic Social Defeat Stress in Different Tests

#### Adolescents’ behavior before and during agonistic interactions with adult males

During the first few days, adolescent males demonstrated very active behavior near the partition, reacting to the adult male in the adjacent compartment. They moved around near the partition, smelled and touched it with one or two paws, climbed onto the partition and hung on it, stuck their noses into the holes. They arranged their nests near the partition. These behaviors were observed during the first few days despite the attacks by an adult male the previous day. After the partitions were removed, the adolescent males approached and followed the adult males even after their attacks. The aggressive adult male in the adjacent compartment was not perceived by adolescent males as a threat, probably because it was unfamiliar (each day an adolescent was encountering a new adult male). After 10 days of exposure to adult males, the behavior of young animals changed: they demonstrated defensive behavior and avoidance in social interactions and most of them (10 of 12 animals) started building their nests in the opposite corner of the cage. Adolescents transported from cage to cage without agonistic interactions with adult males continued to react actively to adult males, trying to climb over the partition and communicate with the neighbor. Most of the adolescents in this group (10 of the 12 animals) built their nests near the partition throughout the experiment. This fact could indicate a lack of inherent negative attitude of adolescents toward adult males.

#### Partition test

One-way ANOVA for repeated measures revealed a significant influence of the factor groups (controls, SDS adolescents, PS adolescents) on the number of approaches (F (2, 30) = 43.34; P<0.001), total time of approaches (F (2, 30) = 21.03; P<0.001), and average time of approaches (F (2, 30) = 22.35; P<0.001); of the factor partner (familiar-unfamiliar) on total time (F (1, 30) = 44.11; P<0.001) and average time (F (1, 30) = 9.23; P<0.005); and of interaction between factors for number of approaches (F (2, 30) = 4.55; P<0.019) and average time (F (2, 30) = 4.91; P<0.014).

**Figure 2:**
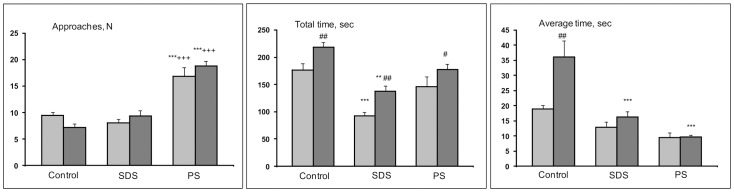
Behavior of adolescents of different experimental groups in the partition test. SDS – social defeat stress; PS – psychological stress; Light columns - familiar partner; dark columns - unfamiliar partner; *P<0.05; **P<0.01; ***P<0.001 *vs* the controls of respective partners; +P<0.05; ++P<0.01; +++P<0.001 *vs* SDS adolescents of respective partners; #P<0.05; ##P<0.01; ###P<0.001 *vs* familiar partner in respective group (n = 10–12 per group).

The number of approaches to the familiar and unfamiliar partner was significantly larger in PS adolescents compared with the respective levels in the controls and SDS adolescents (Fig. 2, results based on the post hoc Bonferroni test, with P<0.001 for all comparisons) Total time spent near the partition, which reflects a reaction to the unfamiliar partner, was significantly longer than reaction to the familiar partner in all groups (P<0.007 for the controls; P<0.024 for the PS adolescents and P<0.003 for the SDS adolescents). Furthermore, compared with the controls, total time spent near the partition was less in SDS adolescents as a reaction to the familiar (P<0.001) and unfamiliar (P<0.002) partners. In the control group, average time of reaction to the unfamiliar partner was higher in comparison with their reaction to the familiar partner (P<0.002). In comparison with the respective controls, average time of reacting to the unfamiliar partner was decreased in the SDS adolescents and PS adolescents (P<0.001 for both groups).

#### Elevated plus-maze test

One-way ANOVA revealed a significant influence of the factor groups (controls, SDS adolescents, PS adolescents) on the number of open-arm entries (F (2, 30) = 4.77; P<0.016); time spent in the center (F (2, 30) = 3.53; P<0.042); closed-arm entry number (F (2, 30) = 5.17; P<0.012) and time (F (2, 30) = 4.97; P<0.014); and the number of peepings (F (2, 30) = 7.98; P<0.002) and head dips (F (2, 30) = 5.71; P<0.008) (Table 1).

**Table 1:** Behavior of adolescents in the elevated plus-maze test.

Parameters	Control	SDS adolescents	PS adolescents
Open arms, N (%)	8,7±2,0	2,9±0,9 *	3,7±1,1 *
Open arms, sec (%)	4,5±1,4	1,1±0,3	2,4±0,9
Center, N (%)	48,8±0,6	44,9±2,3	48,4±0,8
Center, sec (%)	18,0±2,3	8,5±1,8 *	16,9±3,3
Closed arms, N (%)	42,5±2,2	52,2±2,6**	47,8±1,5
Closed arms, sec (%)	77,5±2,5	90,4±2,1*	80,7±3,6
Peepings, N	9,4±1,5	10,8±1,6	4,3±0,5*++
Passages, N	7,5±0,8	8,4±2,0	6,7±1,2
Head dips, N	8,3±1,8	3,5±1,0 *	2,8±0,7**
Total entries	28.8±7,9	24,3 12,4	19,4 10,7

*P<0.05; **P<0.01 *vs* the control; ++P<0.01 *vs* SDS adolescents (n = 10–12 per group).

Compared with the controls, closed-arm entries and time spent there increased (P<0.010 and P<0.014, respectively); based on the post hoc Bonferroni test and percentages of total time spent in the center, numbers of open-arm entries and head dips decreased (P<0.048; P<0.026 and P<0.041, respectively) in SDS adolescents. Percentages of open-arm entries, number of head dips and peepings were decreased (P<0.053, P<0.011, and P<0.017, respectively) in PS adolescents compared with the controls. Number of peepings was significantly lower in PS adolescents compared with SDS adolescents (P<0.002).

#### Social interactions test

One-way ANOVA for repeated measures revealed a significant influence of the factor groups (controls, SDS adolescents, PS adolescents) on the number of approaches to the cylinder (F (2, 31) = 4.61; P<0.018), total time spent near the cylinder (F (2, 31) = 48.88; P<0.001), and number of rearings (F (2, 29) = 7.07; P<0.003). We also found that factor familiar-unfamiliar partners affected the number of approaches to the cylinder (F (1, 31) = 29.33; P<0.001), total time spent near the cylinder (F (1, 31) = 52.59; P<0.001), number of rearings (F (1, 29) = 32.21; P<0.001) and total time of self-grooming (F (1, 29) = 13.71; P<0.001); furthermore, we found interaction effects between factors for the total time spent near the cylinder (F (2, 31) = 20.59; P<0.001) and number of rearings (F (2, 29) = 5.77; P<0.008).

**Figure 3:**
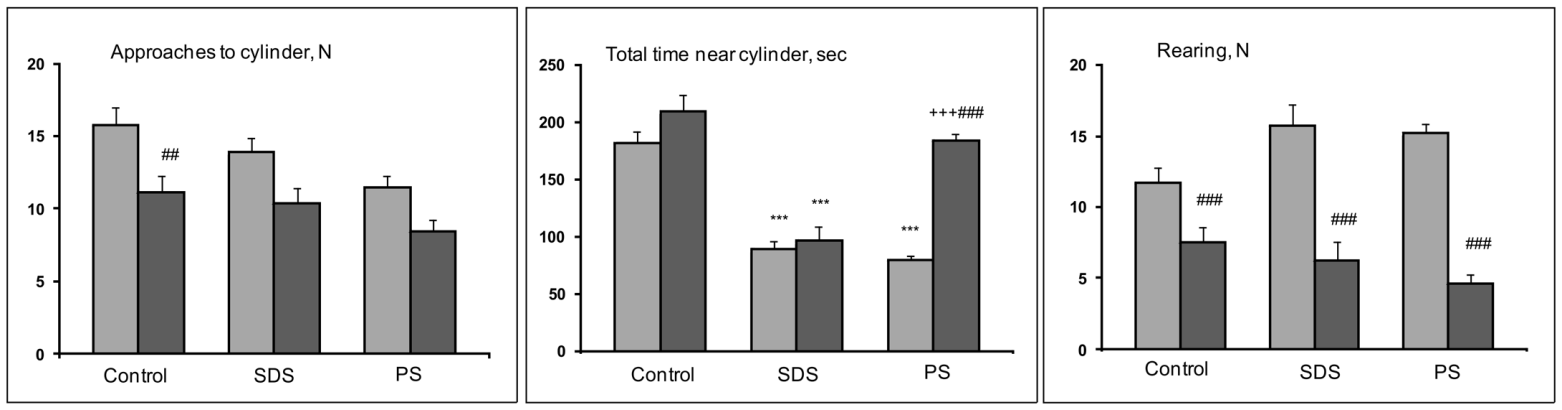
Behavior of adolescents of different experimental groups in the social interactions test. SDS – social defeat stress; PS – psychological stress; Light columns - empty cylinder; dark columns -unfamiliar partner is under cylinder. ***P<0.001 *vs* the controls of respective partners; +++P<0.001 *vs* SDS adolescents of respective partners; ##P<0.01; ###P<0.001 *vs* empty cylinder in respective groups (n = 10–12 per group).

We also found significant differences between the number of approaches to an empty cylinder and a cylinder with a partner in the controls (P<0.006); between the controls and SDS adolescents in the total time spent near an empty cylinder and a cylinder with a partner (P<0.001) and between the controls and PS adolescents (P<0.001); and in the total time spent near an empty cylinder (Fig. 3; all results after Bonferroni test). Additionally, total time spent near a cylinder with partner was increased in the PS adolescents compared with their reaction to an empty cylinder and with SDS adolescents (P<0.001 in both cases). In all groups the number of rearings near the cylinder with a partner was less in comparison with the reaction near an empty cylinder in respective groups (P<0.001 for all groups). There were no significant differences in the number of self-groomings between the groups in all comparisons (P>0.05, data not shown).

#### Open-field test

**Figure 4:**
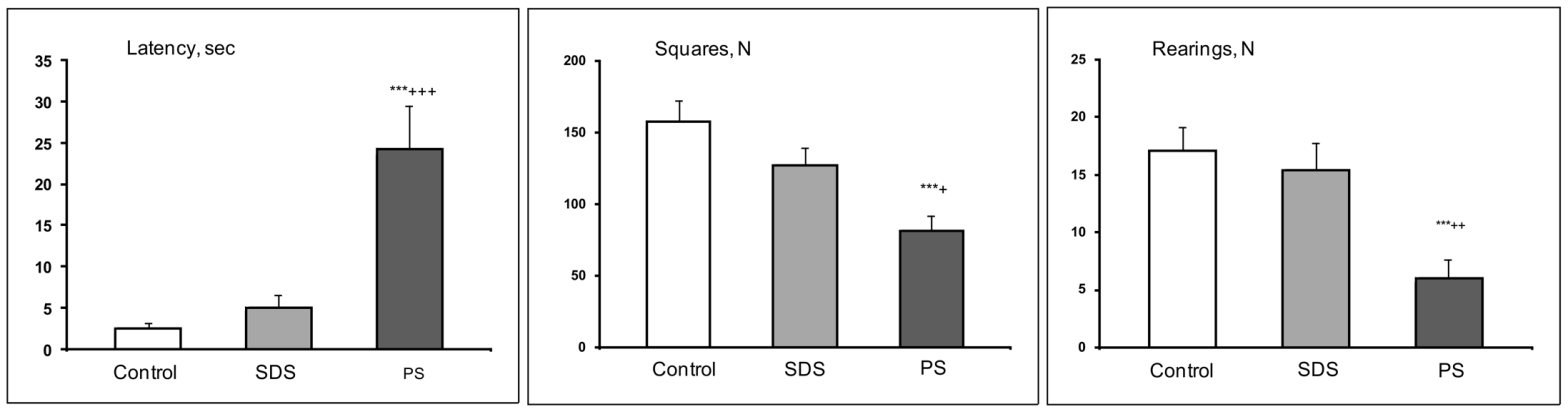
Behavior of adolescents of different experimental groups in the open-field test. SDS – social defeat stress; PS – psychological stress; ***P<0.001 *vs* the controls; +P<0.05; ++P<0.01; +++P<0.001 *vs* SDS adolescents (n = 10–12 per group).

One-way ANOVA revealed a significant influence of the factor groups on the latency time (F (2, 30) = 14.13; P<0.001); number of squares (F (2, 30) = 10.20; P<0.001); and number of rearings (F (2, 30) = 8.36; P≤0.001). Based on the post hoc Bonferroni test, in comparison with the levels in the controls and SDS adolescents, the latency time of first movement increased (for both comparisons P<0.001); number of squares (P<0.001 and P<0.035, respectively) and rearings (P<0.002 and P<0.0095, respectively) decreased in PS adolescents (Fig. 4).

#### Porsolt test

**Figure 5:**
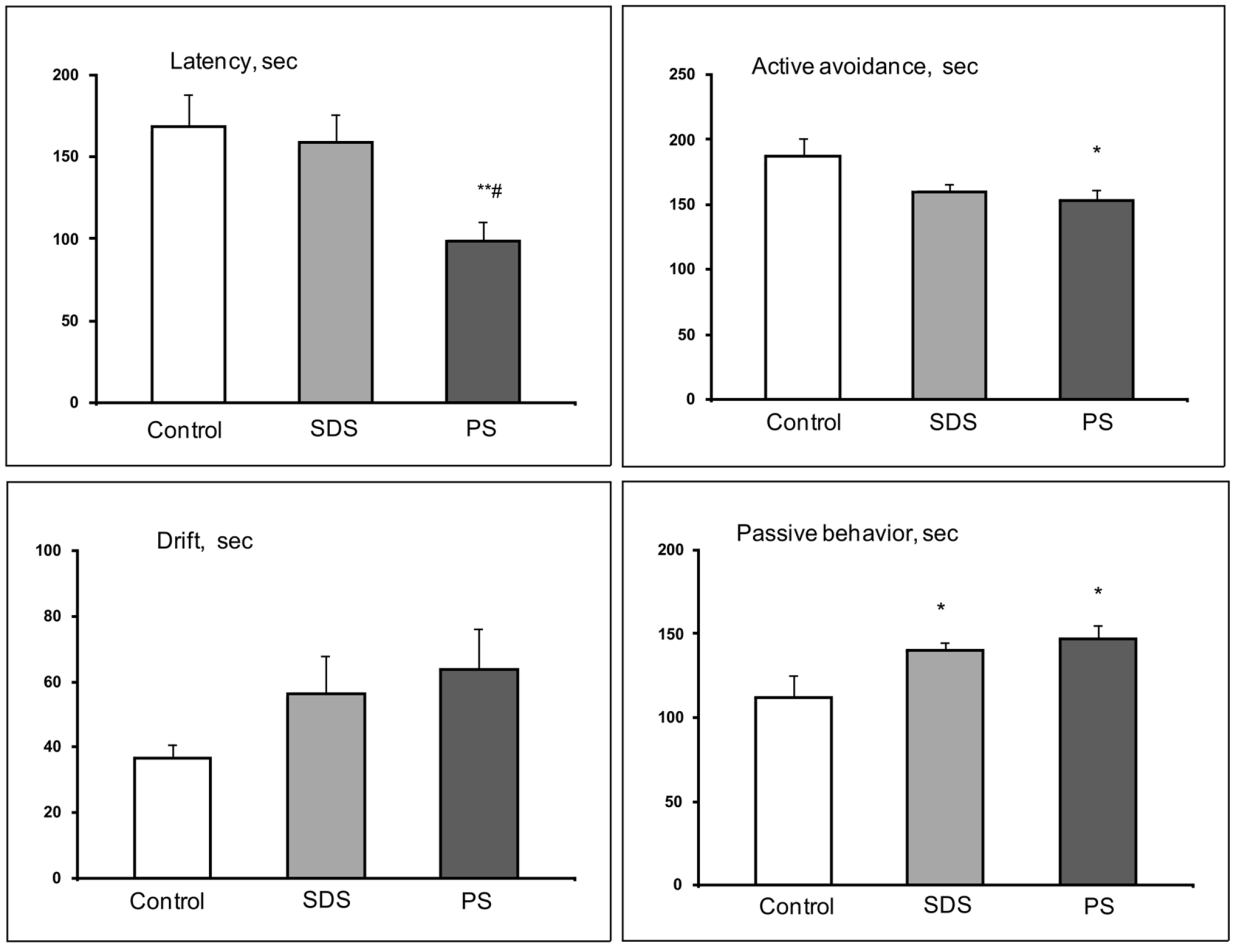
Behavior of adolescents of different experimental groups in the Porsolt test. SDS – social defeat stress; PS – psychological stress; **P<0.01 *vs* the controls. #P<0.05 *vs* SDS adolescents (n = 10–12 per group).

One-way ANOVA revealed a significant influence of the factor groups (controls, SDS adolescents, PS adolescents) on the active avoidance time (F (2, 33) = 3.36; P<0.047), passive behavior time (F (2, 33) = 3.60; P<0.039) and latency time of full immobility (F (2, 32) = 4.64; P<0.017) (Fig. 5). Based on the post hoc LSD test, compared with the level in the controls, active avoidance time decreased in the PS adolescents (P<0.020) and passive behavior time was higher in SDS adolescents (P<0.050) and PS adolescents (P<0.016). Latency time was less in PS adolescents in comparison with the controls and SDS- adolescents (P<0.008 and P<0.020, respectively).

### Behavior of SDS Adolescents after 3 Weeks of Rest

In the partition test, one-way ANOVA for repeated measures revealed a significant influence of the factor partner (familiar-unfamiliar) on the total time (F (1, 21) = 45.07; P<0.001) and average time (F (1, 21) = 35.07; P<0.001) (Table 2). Based on the post hoc Bonferroni test, total time of reaction to the unfamiliar partner was increased in comparison with the reaction to the familiar partner in two groups (P<0.005 for the controls and P<0.001 for SDS adolescents after the rest). Average time as a reaction to unfamiliar partner was increased in SDS adolescents after the rest (P<0.001).

**Table 2:** Behavior of SDS adolescents in behavioral tests after 3 weeks of rest.

Parameters	Control	After the rest
**Partition test**		
Approaches,familiar partner, N	10.6±1.1	10.1±1.0
unfamiliar partner, N	9,7±0.7	8,6±0.9
Total time, familiar partner, sec	127.8±7.8	135.2±16.6
unfamiliar partner, sec	187.4±13.9^#^	219.2±13.0^##^
**Plus-maze test**		
Open arms, N (%)	10.5±1.9	2.8±1.0 ***
Open arms, sec (%)	10.2±2.8	1.2±0.4 **
Center, N (%)	47.6±0.5	47.1±1.2
Center, sec (%)	14,0±1.4	16.3±2.7
Closed arms, N (%)	41.9±2,1	48.6±1.8 *
Closed arms, sec (%)	75.9±3,1	81.6±3.1
Total entries	21.0±2.4	20.3±2.9
Peepings, N	7.2±0,8	7,6±1.0
Passages, N	4.6±0,9	5.3±1.0
Head dips, N	5.5±0,9	3.5±0.7
**Social interactions test**		
Approaches to empty cylinder, N	13.0±0.7	13.7±0.7
- cylinder with partner, N	12.0±0.5	13.7±0.9
Total time -,empty cylinder, sec	103,4±10,7	89,8±5,9
- cylinder with partner, sec	204,3±9,3^##^	189,5±5,0^##^
Rearing - empty cylinder, N	17,0±2.4	17,6±1,6
- cylinder with partner, N	9,8±1,3	11,1±1,8
Self-grooming, empty cylinder, sec	12,4±2,9	13,0±2.6
- cylinder with partner, sec	12,5±3,2	5,6±1,4***
**Open-field test**		
Latency of first movement, sec	2,4±0,8	17,2±6,9*
Crossed squares, N	117,5±10,2	94,1±11,8
Rearing, N	15,6±2,6	11.7±2,5
Self-grooming, sec	23,9±4,9	15,9±3.1
**Porsolt test**		
Latency of first immobility, sec	173,6±19,8	185,9±16,7
Active avoidance, sec	191,1±8,7	193,2±11,3
Drift, sec	39,4±5,9	32,9±7,4
Immobility, sec	69,4±8,6	66,3±9,6
Passive behavior, sec	108,7±8,8	107±11,3
Number of animals	11	12

*P<0.05; **P<0.01; ***P<0.001 *vs* the control; ^#^P<0.01; ^##^P<0.001 *vs* empty cylinder.

**Figure 6:**
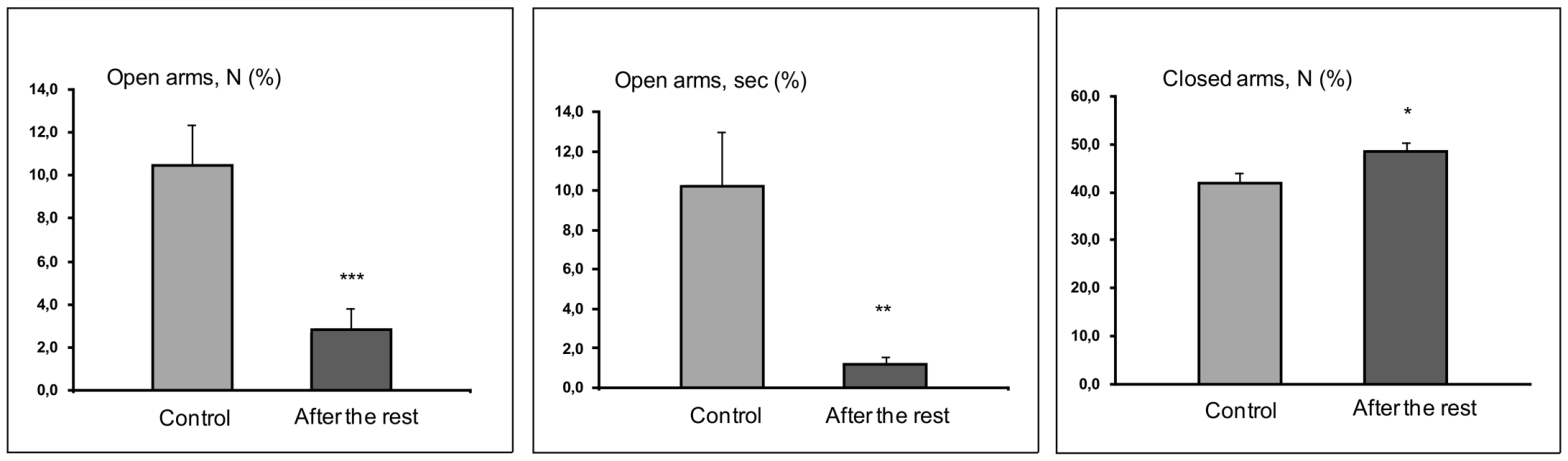
Behavior of adolescents (the controls and group of adolescents after 3 weeks of rest in the elevated plus-maze test. *P<0.05; **P<0.01; ***P<0.001 *vs* the controls (n = 11–12 per group).

In the elevated plus-maze test, percentages of open-arm entries and time spent in open arms were less (t = 3.51; P<0.001 and t = 3.08; P<0.003, respectively) and percentages of closed-arm entries were higher (t = 2.45; P<0.018) in SDS adolescents in their adult life than in the controls (Fig. 6, Table 2). In the open-field test, compared with the respective levels in the controls, latency time of first movement from the center was higher in the adolescents grown to adulthood compared with respective unstressed controls (t = 2.26, P<0.035). Other parameters of plus-maze behaviors, open-field test and all parameters in the Porsolt tests did not differ significantly in the control and SDS adolescents that were stressed in childhood and then received a period of rest.

In the social interactions test, one-way ANOVA for repeated measures revealed a significant influence of factor “partner” (an empty cylinder and a cylinder with partner) on the total time spent near the cylinder with the unfamiliar partner under it (F (1, 20) = 244.32; P<0.001) and on self-grooming time (F (1, 20) = 9.39; P<0.006). Based on the post hoc Bonferroni test, total time near the cylinder with the unfamiliar partner was increased in the controls and grown adolescents after the period of rest as compared with this behavior when the cylinder was empty in respective groups (for both P<0.001).

Percentages of the control mice and grown SDS adolescents of matching age that were winners in first agonistic interactions with group-housing partners were similar (47% (14/30) and 43% (13/30), respectively). However, expression of aggressive behavior in SDS adolescents, which were stressed in childhood, was different (Table 3): in comparison with non-stressed in childhood controls, the latency time of the first attack was significantly shorter (U = 46.5; P<0.031) and the total time of hostile behavior, including attacks, diggings and aggressive grooming, was significantly higher (U = 39.5; P<0.021) in adult SDS adolescents. Total time of attacks differed on the tendency level (U = 55.0; P<0.081). In addition to attacks, 69% (9/13) of grown SDS adolescents and 14% (2/14) of controls demonstrated aggressive grooming (chi-square = 8,43; P<0.004). Total time of digging behavior did not differ in this comparison (P>0.05). Both groups of grown adolescents never demonstrated threats and rotations. In both groups, very strong uncontrollable aggression was noted in animals: 4 males (31%) from adolescents stressed in childhood and 2 males (14.2%) of the controls demonstrated total time of attacks over 260 sec on average.

**Table 3:** Behavior of SDS adolescents in the agonistic interaction test after 3 weeks of rest.

Parameters	Control	Following rest
**Agonistic interaction test (description)**		
Latency time, sec	99,4±20,7	43,9±11,7*
Attacks, N	15,3±4,0	13,9±1,9
Attacks, sec	101,1±22.6	151,2±24,5+
Aggressive grooming, N	14%	69%*
Digging, sec	36,9±6,9	27,2±6,3
Self-grooming, sec	25,1±7,6	25,5±7,7
Hostile behavior, sec	145,8±24,3	192,5±20,2*
Number of animals	14	13

*P<0.05; **P<0.001 vs control; + - tendency P<0.081.

### Division of Neural Progenitors in the Hippocampus

**Figure 7:**
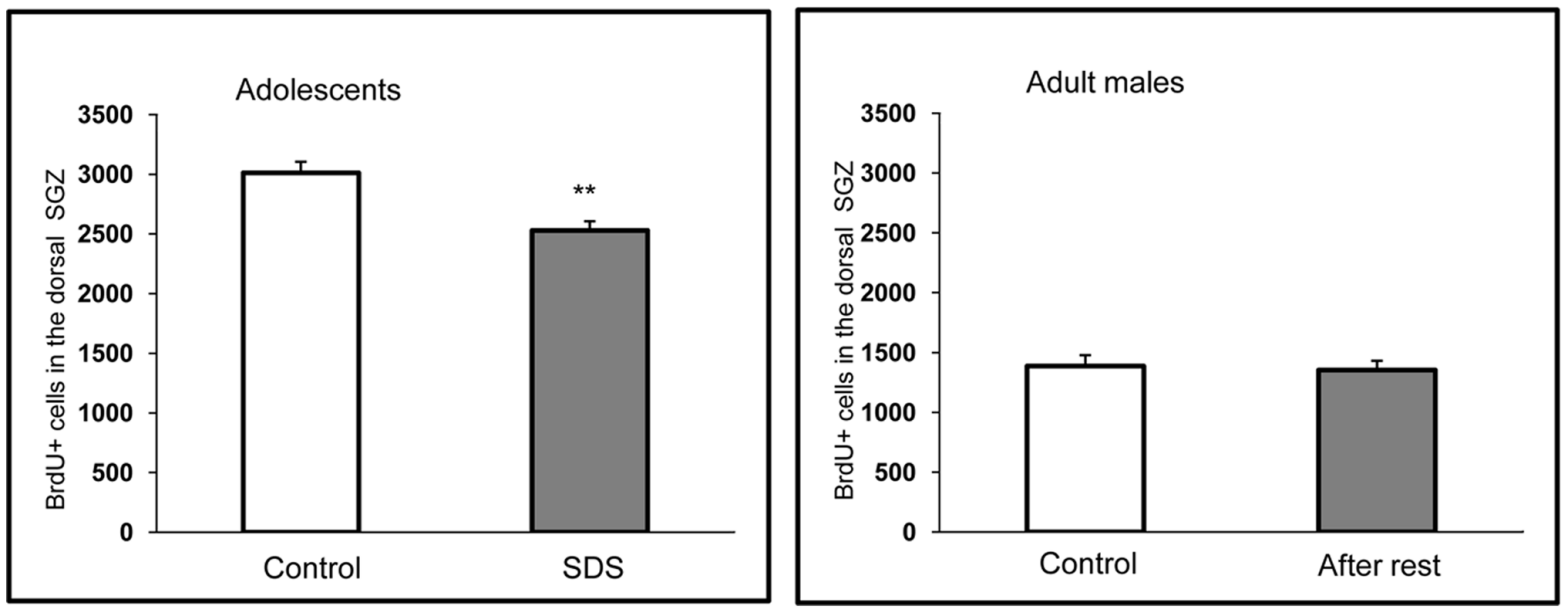
BrdU-positive cells in hippocampal subgranular zone of the dentate gyrus in SDS adolescents and in grown adolescents after 3 weeks of the rest. SDS – social defeat stress **P<0.01 - *vs* the controls (n = 6–7 per group).

Changes in hippocampal neurogenesis correlate with a wide range of behavioral settings [25], [26], [27], [28]. Therefore, we analyzed division of neural stem and progenitor cells in the subgranular zone of the dentate gyrus by labeling dividing cells with a thymidine analog, BrdU. We found that the number of dividing cells was significantly smaller in SDS adolescents than in controls (Fig. 7; P<0.05). Notably, this difference was not detected when animals stressed in adolescence were allowed to rest for 3 weeks.

## Discussion

Together, our results demonstrate that hostile social environment in adolescence compromises the psychoemotional state and social behavior of the animals in adulthood. Considering the psychogenic factors acting on the SDS and PS adolescents in our experimental paradigms, we found that for SDS adolescents, daily agonistic interactions with adult aggressive males resulted in social defeat stress; in other words, hostile social environment was revealed as a critical factor in our experiments**.** Our results also indicate that for PS adolescents, deprivation of the opportunity to communicate with adult males, which can be described as communication deprivation stress (CDS), is a specific psychogenic factor. For both SDS adolescents and PS adolescents, social instability stress (SIS), which resulted from the placement of a young male into an unfamiliar cage with unfamiliar litter, and with an unfamiliar adult partner living on its own territory in the partitioned compartment, is revealed as yet another psychogenic factor. There is growing evidence that social instability, induced by combinations of alterations in cage partners, crowding, social isolation, and maternal separation, could constitute a strong stress [7], [9], [14], [16], [29], [30], [31], [32], [33]. Such stress induces a range of changes in rats and mice, including elevation of corticosterone levels and heightened anxiety-like behavior in juveniles [16], along with persistent impairments in the performance of hippocampal-dependent learning and memory tasks [25]. The results of our study indicate that the combination of stressful factors CDS+SIS as well as SDS+SIS has great impact on the performance of adolescents in some situations. This also indicates that deprivation of communication may be another strong stressful factor for adolescents.

Habitation in a common cage with an aggressive adult male and social defeat as a result of daily agonistic interactions in combination with SIS significantly affects sociability of the SDS adolescents. During the first few days, young males demonstrated high interest in adult males; they built their nests near the partitions and during the activation period spent much time near the partition reacting to the adult males. This behavior appears to be motivated by the drive for communicating with an adult partner. When the partition was removed, the adolescents approached the adult males, sniffed and followed the aggressors even after their attacks, thereby demonstrating absence of fear and anxiety. By the end of 2 weeks, adolescents exposed to SDS stayed mostly near the cage wall opposite the partition during the activation period and built their nests in the corner opposite to the partition. In the agonistic interaction test they avoided aggressors and did not approach them. In SDS adolescents, the level of communicativeness estimated in the home cage in the partition test and in a novel situation in the social interaction test was decreased: they reacted to familiar and unfamiliar partners in the neighboring compartment of the common cage and investigated the cylinder with an unfamiliar partner significantly more rarely that the control mice. This suggests that in SDS adolescents, social communication is disturbed as a result of increased anxiety. This conclusion is supported by the results of the elevated plus-maze test, which show a decrease in the number of entries into the open arms of the maze, a decrease in the total time spent in the center, and an increase in the number of entries and time spent in the closed arms, as well as a decrease in risk assessment parameters (head dips). Notably, in the Porsolt test, SDS adolescents demonstrated a significant increase in the total time of passive behavior compared with the control mice, which is indicative of increased depressiveness or development of helplessness in unavoidable situations. Thus, SDS+SIS leads to profound psychoemotional changes in adolescents, which affect their social and individual behaviors. This conclusion is also supported by studies demonstrating development of increased anxiety after social isolation stress [4] and under social instability stress [30], [33] and depressive-like behavior under SDS [34] or chronic mixed-modality stressors (isolation, restraint and SDS) [12] in adolescent male and female rats.

*Notably, the changes in adolescent behaviors under chronic SDS are similar to those in adult males in similar experimental paradigms. In adult mice, long SDS dramatically increases anxiety and depressiveness, decreases sucrose consumption* [*35*], [36], [*37*] *, disturbs social communications, and increases repetitive behaviors* [*38*] *. However, there are also important distinctions in the response of adults and adolescents to SDS: while in adult males depressiveness was shown to develop after 21 days of SDS in our experimental paradigm* [*35*] *, in SDS adolescents a depressive-like state develops faster, appearing after 14 days in the Porsolt test as longer periods of passive behavior (* *Fig. 5* *). This indicates a higher sensitivity of adolescents to the negative effects of the social stress, compared with the adult animals.*

*Profound behavioral changes induced by SDS in adolescents are supported by the analysis of hippocampal neurogenesis: the number of BrdU-positive cells in the subgranular zone of the dentate gyrus was significantly decreased compared with the controls; this finding parallels the reports of decreased cell division in the dentate gyrus of adult male mice and rats exposed to SDS [39 40, 41, 42,43].*

Interestingly, the behaviors of PS adolescents that were deprived of physical communication with adult partners did not change significantly in the partition and social interaction tests. Such adolescents continued to actively react to an adult male in the home cage, trying to climb over the partition and communicate with the neighbor. The level of communicativeness, estimated by the total time spent near the partition as a reaction to an adult male, was similar to that observed in the controls. The number of approaches to the partition was significantly larger in this comparison. Most adolescents built their nests near the partition throughout the experiment. In a novel situation of social interaction test, these adolescents actively approached the unfamiliar partner in the perforated cylinder. Together, these experiments suggest that social communicativeness of the PS adolescents did not suffer significantly. They also indicate a lack of inherent negative attitude and fear toward adult males and a strong motivation to communicate with them.

In contrast to the manifestations of social communicativeness, PS adolescents demonstrated increased anxiety similar to that observed in SDS adolescents. In the elevated plus-maze test, open-arm entries and risk assessment parameters (peeping and head dips) were significantly lower in the PS adolescents than in the controls. In the aversive situation of the open-field test, a longer latency of the first movements from the central squares as well as a decreased number of crossed squares and rearings were evident in PS adolescents compared with the controls and SDS adolescents. Thus, the transfer from the home cage to the compartment of an unfamiliar cage, which is considered as SIS, in combination with communication deprivation, decreases exploratory and movement activities, increases emotionality, and induces development of a high level of anxiety. In the Porsolt test, PS adolescents displayed a high level of depressiveness similar to that in SDS adolescents: the total time of active behavior was shorter and the total time of passive behavior longer than in the controls. Additionally, PS adolescents demonstrated a shorter latency of full immobility than the controls and SDS adolescents, which could be also interpreted as depressiveness. Thus, aversive conditions of the Porsolt and open-field tests revealed strong negative effects of the combination of CDS+SIS. Some behavioral changes were more pronounced in PS adolescents compared with SDS adolescents that had physical agonistic interactions with the aggressors.

Initially, we planned on using PS adolescents to differentiate the effects of SDS under agonistic interactions from the psychological effects of social instability induced by daily transfer to a novel cage on unfamiliar litter, with an unfamiliar adult male in the adjacent compartment of a partitioned common cage. Detailed analysis of the behavioral motivation in PS and SDS adolescents based on different tests in comparison with each other and with the controls makes it possible to offer additional interpretations. Our behavioral data demonstrate development of increased anxiety and depressiveness in both groups of adolescents compared with the controls. These changes, to some degree, can be attributed to SIS as a psychopathogenic factor that is common to both groups. For the SDS adolescents, daily social defeats inducing the fear of attacks could be regarded as a critical stress factor. Comparison of the results with the control and PS adolescent groups indicates that SDS induces disturbances in communication and a more pronounced level of anxiety. In PS adolescents, the majority of changes were found in open-field behaviors. These changes could produce additional effects of CDS–the lack of opportunities to communicate with conspecifics. An important question is how long do the changes in psychoemotional state induced in SDS adolescents by hostile environment persist. After 2 weeks of agonistic interactions with adult partners, SDS adolescents were placed for rest and for social support. They lived with nonaggressive males of the same age and similar social experience in familiar cages. We found that in such SDS adolescents, most behavioral parameters were restored to the control levels (Table 2). The exceptions were (a) the latency of the first movement from the center in the open-field test, which can be interpreted as increased level of emotionality, and (b) high level of anxiety, estimated by a decrease in the number and time of open-arm entries and an increase in the number of closed-arm entries in the plus-maze test. This indicates a high level of anxiety induced by housing in a hostile environment for at least 3 weeks. Thus, unlike other behavioral parameters, increased anxiety developed in adolescence persists into adult life. Taking into account data confirming interactions between aggression and anxiety in animals [44], [45] and humans [46], it is plausible that anxiety is a major contributor to increasing impulsiveness and aggressiveness demonstrated by animals in provoking situations. In agonistic interactions, adult males that were stressed in adolescence demonstrated short latency of the first attacks and increased time of hostile behavior compared with control male mice. Our results parallel the data obtained with young hamsters exposed daily to aggressive adults: repeated exposure to social stress during puberty alters the development of agonistic behavior in adulthood [47], [48]. Interestingly, while initial social stress decreased division of neural progenitors in the dentate gyrus of the adolescents in our experiment, cell division in the subgranular zone was restored to the control levels in the adults (Fig. 6).

Emerging evidence indicates that social defeats experienced in childhood and adolescence can have different consequences for the behaviors of animals in adulthood. Socially defeated rats demonstrate increased anxiety in adulthood [49]. In contrast, adolescent male rats exposed to social defeat exhibit reduced anxiety and more efficient risk assessment in the elevated plus-maze test as adults [50]. Another study failed to find an effect of social defeats on anxiety in adulthood in female rats, but did find evidence of heightened depressive behavior [12].

Under chronic social defeat stress, adult brain tissue undergoes numerous changes, including changes in gene expression (sometimes leading to long-lasting effects) [35], [51], DNA methylation, histone acetylation and chromatin remodeling [52], [53], and changes in hippocampal neurogenesis [39], [40], [41], [42], [43]. Moreover, stress-induced pathologies may be associated with paternal transmission [54]. Taking these reports and our results into consideration, one would expect similar molecular and cellular changes in the brains of adolescents, which are more vulnerable to stress than adults. Early-life stress can provoke the development of autistic spectrum symptoms [55], and adolescents stressed in childhood can demonstrate markedly disturbed social communication and avoidance of social contact [56], long-term deviations in socialization and communication, as well as inappropriate and often self-destructive social behavior. Therefore, our approach may be useful for studies exploring the consequences of long-term changes that arise in adolescence for subsequent psychoemotional state in adulthood–in particular for the development of autistic spectrum disorders.
